# 3-(9*H*-Carbazol-9-yl)propan-1-ol

**DOI:** 10.1107/S1600536810051809

**Published:** 2010-12-18

**Authors:** N. Haridharan, V. Ramkumar, R. Dhamodharan

**Affiliations:** a Department of Chemistry, IIT Madras, Chennai, TamilNadu, India

## Abstract

In the title compound, C_15_H_15_NO, the dihedral angle between the benzene rings is 2.25 (2)°. The C—C—C—O atoms of the propanol side chain are in a *gauche* conformation [torsion angle = −60.5 (2)°]. In the crystal, O—H⋯O hydrogen bonds link the mol­ecules into *C*(2) chains propagating in [100]. The O-bonded H atom is disordered over two sites of equal occupancy.

## Related literature

For applications of the title compound, see: Chakkaravarthi *et al.* (2008 ▶); Murugavel *et al.* (2009 ▶). For related structures, see: Chen *et al.* (2009 ▶); Uludağ *et al.* (2010 ▶)
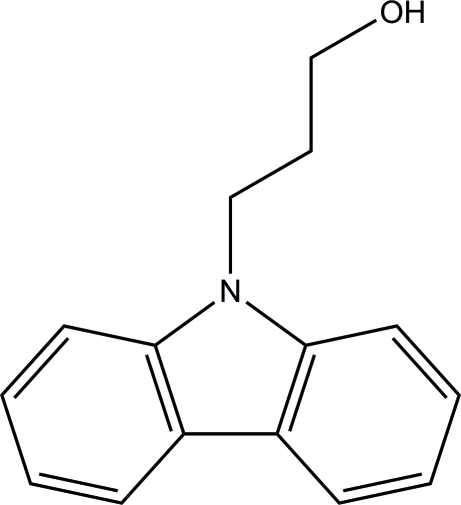


## Experimental

### 

#### Crystal data

C_15_H_15_NO
*M* *_r_* = 225.28
Monoclinic, 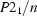

*a* = 5.2930 (6) Å
*b* = 12.5935 (16) Å
*c* = 17.954 (2) Å
β = 97.778 (6)°
*V* = 1185.8 (3) Å^3^
*Z* = 4
Mo *K*α radiation
μ = 0.08 mm^−1^
*T* = 298 K
0.32 × 0.20 × 0.18 mm

#### Data collection

Bruker APEXII CCD diffractometer
Absorption correction: multi-scan (*SADABS*; Bruker, 2004 ▶) *T*_min_ = 0.975, *T*_max_ = 0.986
8147 measured reflections
2721 independent reflections
1566 reflections with *I* > 2σ(*I*)
*R*_int_ = 0.041

#### Refinement

*R*[*F* ^2^ > 2σ(*F* ^2^)] = 0.055
*wR*(*F* ^2^) = 0.142
*S* = 0.97
2721 reflections
162 parameters
1 restraint
H atoms treated by a mixture of independent and constrained refinement
Δρ_max_ = 0.16 e Å^−3^
Δρ_min_ = −0.21 e Å^−3^

### 

Data collection: *APEX2* (Bruker, 2004 ▶); cell refinement: *SAINT* (Bruker, 2004 ▶); data reduction: *SAINT*; program(s) used to solve structure: *SHELXS97* (Sheldrick, 2008 ▶); program(s) used to refine structure: *SHELXL97* (Sheldrick, 2008 ▶); molecular graphics: *ORTEP-3* (Farrugia, 1997 ▶); software used to prepare material for publication: *SHELXL97*.

## Supplementary Material

Crystal structure: contains datablocks global, I. DOI: 10.1107/S1600536810051809/hb5760sup1.cif

Structure factors: contains datablocks I. DOI: 10.1107/S1600536810051809/hb5760Isup2.hkl

Additional supplementary materials:  crystallographic information; 3D view; checkCIF report

## Figures and Tables

**Table 1:** Hydrogen-bond geometry (Å, °)

*D*—H⋯*A*	*D*—H	H⋯*A*	*D*⋯*A*	*D*—H⋯*A*
O1—H1*O*⋯O1^i^	0.95 (2)	1.91 (3)	2.834 (3)	163 (6)
O1—H1*OA*⋯O1^ii^	0.89 (6)	1.96 (6)	2.850 (4)	172 (6)

Symmetry codes: (i) 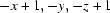; (ii) 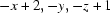.

**Fig. 1.**
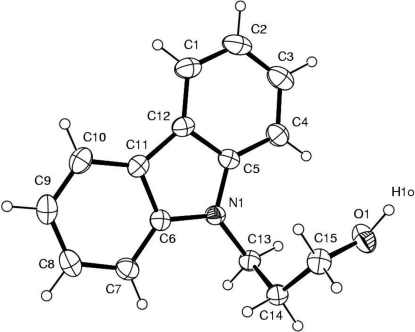
View of (I) with atoms represented as 30% probability ellipsoids.

**Fig. 2.**
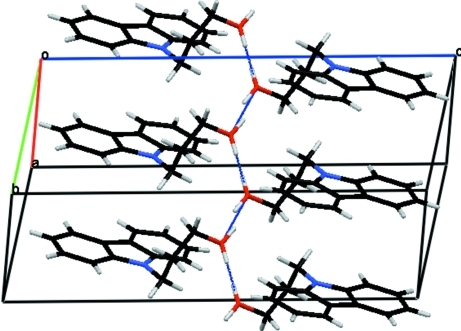
The packing diagram showing the O—H···O interaction along the a axis.

## supplementary crystallographic information

### Comment

The title compound, C_15_H_15_NO, (I) is a carbazole based alcohol derivative. It has a tricyclic structure, consisting of two six-membered benzene ring fused on either side of a five-membered nitrogen-containing ring (Uludağ *et al.* 2010; Chen *et al.* 2009). A propan-1-ol group is linked to the nitrogen atom of the carbazole.

It is an important heterocyclic aromatic compound in which the alcohol group is used as a linker for the preparation of various carbazole derivatives (Murugavel *et al.* 2009; Chakkaravarthi *et al.* 2008). The tricyclic structure is essentially planar, making a dihedral angle of 2.25 (2)° with the two outer most aromatic rings. The crystal packing is stabilized by a bifurcated O—H···O interaction linking the molecules into chains along the *a* axis.

### Experimental

Carbazole (5 g, 0.03 moles), sodium hydride (2.88 g, 0.12 moles) and dry THF (400 ml) were placed in a 3-neck round bottomed flask equipped with a magnetic stirrer. The flask was purged with dry N_2_ gas, sealed and placed in a salt ice-bath (-15 °C). The reaction mixture was allowed to stir for 1 h. 3-bromo-1-propanol (4 g, 0.03 mole), was then added slowly to the reaction mixture through a syringe. The reaction was allowed to continue for a period of 12 h, at ambient temperature. The product obtained was isolated by quenching the excess sodium hydride and the solvent was evaporated. The final product was dissolved in ethyl acetate, rinsed with water and dried with anhydrous MgSO_4_. The product was purified by column chromatography technique using 10% ethyl acetate in hexane as the eluent to obtain pure bright white crystals. Recrystallization of the compound from chloroform gave colourless blocks of (I).

### Refinement

The oxygen H atom was located in a diference Fourier map and refined isotropically. Other hydrogen atoms were fixed geometrically and allowed to ride on the parent carbon atoms, with aromatic C—H = 0.93 Å and methylene C—H = 0.97 Å. The displacement parameters were set for phenyl H atoms at *U*_iso_(H) = 1.2*U*_eq_(C)and methylene H atoms at *U*_iso_ (H) = 1.2*U*_eq_(C). The oxygen H atom is disordered in two orientations.

### Crystal data

**Table:**

C_15_H_15_NO	*F*(000) = 480
*M**_r_* = 225.28	*D*_x_ = 1.262 Mg m^−^^3^
Monoclinic, *P*2_1_/*n*	Mo *K*α radiation, λ = 0.71073 Å
Hall symbol: -P 2yn	Cell parameters from 1647 reflections
*a* = 5.2930 (6) Å	θ = 2.3–23.5°
*b* = 12.5935 (16) Å	µ = 0.08 mm^−^^1^
*c* = 17.954 (2) Å	*T* = 298 K
β = 97.778 (6)°	Block, colourless
*V* = 1185.8 (3) Å^3^	0.32 × 0.20 × 0.18 mm
*Z* = 4	

### Data collection

**Table:**

Bruker APEXII CCD diffractometer	2721 independent reflections
Radiation source: fine-focus sealed tube	1566 reflections with *I* > 2σ(*I*)
graphite	*R*_int_ = 0.041
phi and ω scans	θ_max_ = 28.4°, θ_min_ = 2.8°
Absorption correction: multi-scan (*SADABS*; Bruker, 2004)	*h* = −5→7
*T*_min_ = 0.975, *T*_max_ = 0.986	*k* = −16→16
8147 measured reflections	*l* = −23→23

### Refinement

**Table:**

Refinement on *F*^2^	Primary atom site location: structure-invariant direct methods
Least-squares matrix: full	Secondary atom site location: difference Fourier map
*R*[*F*^2^ > 2σ(*F*^2^)] = 0.055	Hydrogen site location: inferred from neighbouring sites
*wR*(*F*^2^) = 0.142	H atoms treated by a mixture of independent and constrained refinement
*S* = 0.97	*w* = 1/[σ^2^(*F*_o_^2^) + (0.0591*P*)^2^ + 0.2606*P*] where *P* = (*F*_o_^2^ + 2*F*_c_^2^)/3
2721 reflections	(Δ/σ)_max_ < 0.001
162 parameters	Δρ_max_ = 0.16 e Å^−^^3^
1 restraint	Δρ_min_ = −0.21 e Å^−^^3^

### Special details

**Table:**

Geometry. All e.s.d.'s (except the e.s.d. in the dihedral angle between two l.s. planes) are estimated using the full covariance matrix. The cell e.s.d.'s are taken into account individually in the estimation of e.s.d.'s in distances, angles and torsion angles; correlations between e.s.d.'s in cell parameters are only used when they are defined by crystal symmetry. An approximate (isotropic) treatment of cell e.s.d.'s is used for estimating e.s.d.'s involving l.s. planes.
Refinement. Refinement of *F*^2^ against ALL reflections. The weighted *R*-factor *wR* and goodness of fit *S* are based on *F*^2^, conventional *R*-factors *R* are based on *F*, with *F* set to zero for negative *F*^2^. The threshold expression of *F*^2^ > σ(*F*^2^) is used only for calculating *R*-factors(gt) *etc*. and is not relevant to the choice of reflections for refinement. *R*-factors based on *F*^2^ are statistically about twice as large as those based on *F*, and *R*- factors based on ALL data will be even larger.

### Fractional atomic coordinates and isotropic or equivalent isotropic displacement parameters (Å^2^)

**Table:**

	*x*	*y*	*z*	*U*_iso_*/*U*_eq_	Occ. (<1)
C1	0.2244 (4)	0.30270 (16)	0.27538 (14)	0.0562 (6)	
H1	0.1063	0.3269	0.2360	0.067*	
C2	0.2145 (5)	0.33733 (17)	0.34732 (16)	0.0648 (7)	
H2	0.0895	0.3857	0.3565	0.078*	
C3	0.3882 (5)	0.30124 (17)	0.40636 (14)	0.0630 (7)	
H3	0.3773	0.3259	0.4546	0.076*	
C4	0.5764 (4)	0.23001 (16)	0.39565 (12)	0.0519 (6)	
H4	0.6922	0.2059	0.4357	0.062*	
C5	0.5869 (3)	0.19539 (14)	0.32266 (11)	0.0395 (5)	
C6	0.6937 (3)	0.11621 (14)	0.21952 (11)	0.0389 (5)	
C7	0.8142 (4)	0.05761 (15)	0.16897 (12)	0.0484 (5)	
H7	0.9554	0.0155	0.1851	0.058*	
C8	0.7165 (5)	0.06429 (17)	0.09406 (12)	0.0562 (6)	
H8	0.7949	0.0265	0.0590	0.067*	
C9	0.5048 (5)	0.12569 (17)	0.06958 (13)	0.0589 (6)	
H9	0.4426	0.1280	0.0186	0.071*	
C10	0.3857 (4)	0.18309 (16)	0.11946 (12)	0.0527 (6)	
H10	0.2430	0.2239	0.1026	0.063*	
C11	0.4806 (3)	0.17964 (14)	0.19561 (11)	0.0407 (5)	
C12	0.4124 (4)	0.23114 (14)	0.26192 (11)	0.0412 (5)	
C13	0.9639 (3)	0.06950 (15)	0.34094 (11)	0.0429 (5)	
H13A	1.0058	0.1062	0.3885	0.051*	
H13B	1.1125	0.0724	0.3148	0.051*	
C14	0.9059 (4)	−0.04550 (14)	0.35637 (11)	0.0436 (5)	
H14A	0.8677	−0.0822	0.3087	0.052*	
H14B	1.0576	−0.0778	0.3834	0.052*	
C15	0.6889 (4)	−0.06224 (17)	0.40063 (12)	0.0502 (5)	
H15A	0.5344	−0.0327	0.3732	0.060*	
H15B	0.6623	−0.1378	0.4067	0.060*	
N1	0.7554 (3)	0.12450 (12)	0.29658 (9)	0.0406 (4)	
O1	0.7366 (3)	−0.01369 (15)	0.47257 (9)	0.0653 (5)	
H1O	0.594 (9)	−0.009 (6)	0.500 (4)	0.13 (2)*	0.50
H1OA	0.903 (11)	−0.012 (5)	0.489 (4)	0.10 (2)*	0.50

### Atomic displacement parameters (Å^2^)

**Table:**

	*U*^11^	*U*^22^	*U*^33^	*U*^12^	*U*^13^	*U*^23^
C1	0.0484 (13)	0.0443 (11)	0.0776 (18)	0.0071 (10)	0.0148 (12)	0.0099 (11)
C2	0.0610 (15)	0.0465 (12)	0.093 (2)	0.0095 (11)	0.0321 (15)	−0.0026 (13)
C3	0.0699 (16)	0.0525 (13)	0.0715 (17)	−0.0036 (13)	0.0278 (14)	−0.0149 (12)
C4	0.0557 (13)	0.0503 (12)	0.0503 (14)	−0.0030 (11)	0.0098 (10)	−0.0076 (10)
C5	0.0368 (10)	0.0359 (9)	0.0471 (12)	−0.0044 (9)	0.0107 (9)	−0.0025 (9)
C6	0.0355 (10)	0.0389 (10)	0.0431 (12)	−0.0057 (9)	0.0083 (9)	0.0016 (9)
C7	0.0475 (12)	0.0470 (11)	0.0528 (13)	−0.0013 (10)	0.0140 (10)	−0.0017 (10)
C8	0.0688 (15)	0.0580 (13)	0.0448 (13)	−0.0106 (12)	0.0180 (11)	−0.0064 (11)
C9	0.0740 (16)	0.0613 (13)	0.0401 (13)	−0.0118 (13)	0.0027 (11)	0.0020 (11)
C10	0.0504 (12)	0.0521 (12)	0.0533 (14)	−0.0071 (10)	−0.0010 (10)	0.0114 (11)
C11	0.0364 (11)	0.0382 (10)	0.0469 (12)	−0.0067 (9)	0.0040 (9)	0.0061 (9)
C12	0.0369 (10)	0.0338 (9)	0.0538 (13)	−0.0016 (9)	0.0088 (9)	0.0057 (9)
C13	0.0290 (10)	0.0519 (11)	0.0473 (12)	−0.0002 (9)	0.0035 (9)	0.0020 (9)
C14	0.0404 (11)	0.0466 (11)	0.0439 (12)	0.0040 (9)	0.0059 (9)	0.0008 (9)
C15	0.0401 (12)	0.0574 (12)	0.0524 (14)	−0.0080 (10)	0.0034 (10)	0.0019 (10)
N1	0.0378 (9)	0.0430 (8)	0.0408 (10)	0.0041 (7)	0.0043 (7)	−0.0014 (7)
O1	0.0512 (11)	0.0989 (13)	0.0473 (10)	−0.0058 (10)	0.0125 (8)	−0.0059 (9)

### Geometric parameters (Å, °)

**Table:**

C1—C2	1.371 (3)	C9—C10	1.369 (3)
C1—C12	1.387 (3)	C9—H9	0.9300
C1—H1	0.9300	C10—C11	1.392 (3)
C2—C3	1.383 (3)	C10—H10	0.9300
C2—H2	0.9300	C11—C12	1.444 (3)
C3—C4	1.373 (3)	C13—N1	1.447 (2)
C3—H3	0.9300	C13—C14	1.514 (2)
C4—C5	1.389 (3)	C13—H13A	0.9700
C4—H4	0.9300	C13—H13B	0.9700
C5—N1	1.388 (2)	C14—C15	1.497 (3)
C5—C12	1.405 (3)	C14—H14A	0.9700
C6—N1	1.382 (2)	C14—H14B	0.9700
C6—C7	1.390 (3)	C15—O1	1.420 (3)
C6—C11	1.401 (3)	C15—H15A	0.9700
C7—C8	1.377 (3)	C15—H15B	0.9700
C7—H7	0.9300	O1—H1O	0.95 (2)
C8—C9	1.383 (3)	O1—H1OA	0.89 (6)
C8—H8	0.9300		
			
C2—C1—C12	119.4 (2)	C10—C11—C6	119.15 (18)
C2—C1—H1	120.3	C10—C11—C12	134.31 (18)
C12—C1—H1	120.3	C6—C11—C12	106.52 (17)
C1—C2—C3	120.8 (2)	C1—C12—C5	118.99 (19)
C1—C2—H2	119.6	C1—C12—C11	134.5 (2)
C3—C2—H2	119.6	C5—C12—C11	106.53 (16)
C4—C3—C2	121.8 (2)	N1—C13—C14	113.60 (15)
C4—C3—H3	119.1	N1—C13—H13A	108.8
C2—C3—H3	119.1	C14—C13—H13A	108.8
C3—C4—C5	117.3 (2)	N1—C13—H13B	108.8
C3—C4—H4	121.3	C14—C13—H13B	108.8
C5—C4—H4	121.3	H13A—C13—H13B	107.7
N1—C5—C4	129.05 (19)	C15—C14—C13	114.89 (16)
N1—C5—C12	109.24 (17)	C15—C14—H14A	108.5
C4—C5—C12	121.72 (18)	C13—C14—H14A	108.5
N1—C6—C7	128.79 (18)	C15—C14—H14B	108.5
N1—C6—C11	109.58 (16)	C13—C14—H14B	108.5
C7—C6—C11	121.62 (19)	H14A—C14—H14B	107.5
C8—C7—C6	117.4 (2)	O1—C15—C14	111.54 (16)
C8—C7—H7	121.3	O1—C15—H15A	109.3
C6—C7—H7	121.3	C14—C15—H15A	109.3
C7—C8—C9	121.7 (2)	O1—C15—H15B	109.3
C7—C8—H8	119.1	C14—C15—H15B	109.3
C9—C8—H8	119.1	H15A—C15—H15B	108.0
C10—C9—C8	120.8 (2)	C6—N1—C5	108.11 (15)
C10—C9—H9	119.6	C6—N1—C13	125.06 (15)
C8—C9—H9	119.6	C5—N1—C13	126.82 (16)
C9—C10—C11	119.3 (2)	C15—O1—H1O	116 (5)
C9—C10—H10	120.4	C15—O1—H1OA	111 (5)
C11—C10—H10	120.4	H1O—O1—H1OA	130 (7)
			
C12—C1—C2—C3	−0.4 (3)	C4—C5—C12—C1	−0.1 (3)
C1—C2—C3—C4	0.1 (3)	N1—C5—C12—C11	−0.46 (19)
C2—C3—C4—C5	0.2 (3)	C4—C5—C12—C11	179.76 (16)
C3—C4—C5—N1	−179.93 (18)	C10—C11—C12—C1	1.0 (4)
C3—C4—C5—C12	−0.2 (3)	C6—C11—C12—C1	179.4 (2)
N1—C6—C7—C8	−178.94 (18)	C10—C11—C12—C5	−178.9 (2)
C11—C6—C7—C8	0.1 (3)	C6—C11—C12—C5	−0.46 (19)
C6—C7—C8—C9	−0.8 (3)	N1—C13—C14—C15	−61.9 (2)
C7—C8—C9—C10	0.6 (3)	C13—C14—C15—O1	−60.5 (2)
C8—C9—C10—C11	0.3 (3)	C7—C6—N1—C5	177.62 (18)
C9—C10—C11—C6	−0.9 (3)	C11—C6—N1—C5	−1.5 (2)
C9—C10—C11—C12	177.4 (2)	C7—C6—N1—C13	−1.3 (3)
N1—C6—C11—C10	179.95 (16)	C11—C6—N1—C13	179.56 (16)
C7—C6—C11—C10	0.7 (3)	C4—C5—N1—C6	−179.01 (18)
N1—C6—C11—C12	1.23 (19)	C12—C5—N1—C6	1.22 (19)
C7—C6—C11—C12	−178.00 (16)	C4—C5—N1—C13	−0.1 (3)
C2—C1—C12—C5	0.4 (3)	C12—C5—N1—C13	−179.89 (16)
C2—C1—C12—C11	−179.4 (2)	C14—C13—N1—C6	−78.8 (2)
N1—C5—C12—C1	179.64 (15)	C14—C13—N1—C5	102.5 (2)

### Hydrogen-bond geometry (Å, °)

**Table:**

*D*—H···*A*	*D*—H	H···*A*	*D*···*A*	*D*—H···*A*
O1—H1O···O1^i^	0.95 (2)	1.91 (3)	2.834 (3)	163 (6)
O1—H1OA···O1^ii^	0.89 (6)	1.96 (6)	2.850 (4)	172 (6)

Symmetry codes: (i) −*x*+1, −*y*, −*z*+1; (ii) −*x*+2, −*y*, −*z*+1.
